# Genetic differences between smokers and never-smokers with lung cancer

**DOI:** 10.3389/fimmu.2023.1063716

**Published:** 2023-02-02

**Authors:** Piotr Kuśnierczyk

**Affiliations:** Laboratory of Immunogenetics and Tissue Immunology, Hirszfeld Institute of Immunology and Experimental Therapy, Polish Academy of Sciences, Wrocław, Poland

**Keywords:** lung cancer, genetics, immunology, ethnic differences, sex/gender differences, therapy

## Abstract

Smoking is a major risk factor for lung cancer, therefore lung cancer epidemiological trends reflect the past trends of cigarette smoking to a great extent. The geographic patterns in mortality closely follow those in incidence. Although lung cancer is strongly associated with cigarette smoking, only about 15% of smokers get lung cancer, and also some never-smokers develop this malignancy. Although less frequent, lung cancer in never smokers is the seventh leading cause of cancer deaths in both sexes worldwide. Lung cancer in smokers and never-smokers differs in many aspects: in histological types, environmental factors representing a risk, and in genes associated with this disease. In this review, we will focus on the genetic differences between lung cancer in smokers versus never-smokers: gene expression, germ-line polymorphisms, gene mutations, as well as ethnic and gender differences. Finally, treatment options for smokers and never-smokers will be briefly reviewed.

*Saru mo ki kara ochiru* [(*Even monkeys fall from trees*; *a Japanese proverb* (1)]

## 1 Introduction

Lung cancer is the leading cause of cancer mortality globally. In 2019, there were almost 24 million cancer cases worldwide, and 10 million deaths, of which 15-20% were caused by lung cancer (2, 3). In 2020, lung cancer occurred in 2.2 million people and resulted in 1.8 million deaths (4) which shows that most patients eventually die of the disease, the ratio of mortality to incidence being 0.87 (5).

Lung cancer, according to histological type, is divided into two broad categories: non-small cell lung carcinoma (NSCLC), the majority of cases, and small cell lung carcinoma (SCLC). NSCLC is, in turn, classified as adenocarcinoma, squamous cell carcinoma, and large cell carcinoma, which differ in therapy requirements and outcome (6). These histological types differ in smoking and smoking cessation effects on lung cancer risk: in the Nurses’ Health Study on 1,062 women with lung cancer, increasing smoking duration was associated with a higher increase in the risk of small cell and squamous cell carcinoma than other histological types of lung cancer, while smoking cessation decreased most strongly the risk of small cell carcinoma (7). Lung adenocarcinoma has been further divided into the terminal transpiratory unit (TRU, or bronchioid), the proximal-inflammatory (PI, squamoid), and the proximal-proliferative (PP, magnoid) subtypes based on gene transcription. These subtypes differ in prognosis and response to therapy (8, 9). Detection and classification of lung cancers is now becoming facilitated by computer techniques including Artificial Intelligence (10–12).

Cigarette smoking as a risk factor of lung cancer was first described more than 70 years ago (13–15). In those years it was predominantly a man’s disease, as much fewer women than men smoked at that time (15). In many countries, the peak of the smoking-related lung cancer epidemic was reached by generations born in the 1930–1940s. Recently, with increasing numbers of female smokers, we observe increasing numbers of deaths of women from lung cancer per 100 000 people in many countries, particularly in Europe, including Poland. Simultaneously, this growth is associated with a decrease in lung cancer death among men in the same countries (16). In Poland, ever-smokers accounted for 51.8% (25.8% former smokers and 26% present smokers) of the adult population in 2019. These values were different for men (32% and 31%, respectively) and women (20.3 and 21.3%) (17, 18). Both the number of cigarettes per day (expressed in packyears) and duration of smoking influence lung cancer risk, even after smoking cessation. Almost all cases of SCLC, squamous cell carcinoma and large cell carcinoma are due to long-term tobacco smoking. Lung adenocarcinoma, although also more frequent in smokers due to higher numbers of smokers among lung cancer patients, occurs also in never-smokers and is the prevalent histological type in this group. As the smoking rate in developed countries decreases, not only the percentage of never-smokers but also the absolute numbers of lung cancer cases in never-smokers is rising, the reason for which is not clear (19) but may be caused by increasing air pollution. Never-smokers with lung cancer have a longer 5-year survival rate than smokers, and those with a smoking history of > 20 pack-years have lower survival rate than those with lower pack-years value (20). Risk factors contributing to lung cancerogenesis in never-smokers may exert an additional, synergistic effect with tobacco smoke in smokers, increasing the probability of cancer in these individuals (21).

Never-smokers are devoid of the strongest cancerogenic agent, tobacco smoking, except for second-hand smoke. However, even these latter patients were not similar to smokers in mutation burden, although some mutations in passive smokers may appear below the detection level (22). As these authors write, “Second-hand tobacco smoke has been causally linked to lung cancer, but it is a weak carcinogen compared to active smoking” (22). Studies on the effects of second-hand smoke are usually less precise, as it is difficult in this case to measure quantitatively the exposure to smoke. Exposure in public places is evaluated simply by asking if respondents saw people smoking. Limiting the analysis to exposure at home gives a possibility of more quantitative establishment of a spouse’s or other cohabitant’s smoking (23).

Environmental factors, in addition to second-hand smoke and stronger from it, contributing to lung cancerogenesis in never-smokers are multiple: exposure to occupational carcinogens, outdoor pollution, X-ray radiation and radon (19). In Asia, most women with lung cancer are never-smokers; however, they are frequently exposed to a household coal combustion. Particularly high frequency of lung cancer, one of the highest in the world, is observed in the Xuanwei/Fuyuan areas of the Yunnan Province, China; it frequently shows familial aggregation (24–27). Childhood exposure to high household coal smoke levels, even after later move to lower exposure place, had much higher contribution to lung cancer risk than the other way round, and tobacco smoking had small additional effect in coal smoke heavily exposed individuals (28). The bituminous coal mined in the Xuanwei area is releasing large amounts of toxic smoke. In the traditional agrarian society in this region women are exceptionally highly exposed because of cooking with unvented fire pits (29). Gene expression, mutations and associations of gene polymorphisms in these, mostly tobacco never-smoking women with lung cancer differ from those in control, cancer-free women, and from smokers with lung cancer, mostly men (see next sections).

Chronic inflammation increases lung cancer risk by raising the chance of mutations, by blocking apoptosis, and by increasing angiogenesis (30). Normally, inflammation induced by irritant agents or infection, after acute and subacute phases, ends with resolution. However, sometimes this resolution phase is not reached, resulting in persistent lung inflammation, which may lead to cancerogenesis (30–32).

Many proteins are engaged in multiple cell activation pathways responsible for cell proliferation and malignant transformation (33, 34). There are so many differences between lung cancer in smokers and never-smokers - in gene expression, genetic polymorphisms and mutations in tumor cells – that it was proposed that lung cancer in these two groups of patients are distinct diseases from genomic and molecular point of view (35–37).

## 2 Gene expression

Strong differences between smokers and never-smokers in gene expression patterns in lung cancer were observed. Thus, a significantly higher expression in adenocarcinoma of *EGFR*, *Ki-67* and *HTERT* in smokers, and *p-AKT* and *p27* in never-smokers was described (38), but numbers of patients in that study were relatively low.

Using RNA microarrays, Powell et al. (39) found that the noncancerous lungs of smokers already had many alterations in gene expression, not seen in nonmalignant lungs of never-smoking patients. Four times as many genes changed their expression in the transition between nonmalignant lung tissue and tumor in never-smokers as in smokers. This suggests that tumors in smokers arise in already genetically altered tissue, while in never-smokers cancerogenesis may start in normal tissue and requires more changes in gene expression. Thus, smoking “prepares” lung tissue for tumor formation, which may explain the much higher frequency of smokers over never-smokers among lung cancer patients.

Examining long noncoding RNAs (lncRNAs) interacting with mRNAs and micro-RNAs, Li et al. (40) identified differentially expressed RNAs of these three categories, constructed a network of competing endogenous RNAs and established a seven-lncRNA prognostic signature which, together with the traditional TNM system, gave superior performance in predicting the patients’ overall survival compared to the clinical model with the TNM staging system only. In patients with stage I/II NSCLC after surgery, Ma et al. (41), using a bioinformatic algorithm, identified 15 survival-related gene transcripts and classified the patients as high risk or low risk in relation to this expression. Again, the combined model of the fifteen-mRNA signature and tumor stage had a higher precision in survival prediction than the tumor stage alone. Unfortunately, smoking status was not included in the analyses by Li et al. (40) and Ma et al. (41) mentioned above. It may be fruitful if their approaches will be applied to the analysis of cohorts containing information about smoking.

Recent multi-omic study on familial lung cancer in Xuanwei area in Yunnan, China, mentioned in *Introduction* as a region with very high lung cancer prevalence among never-smoking women, revealed that smoking and indoor air pollution from the coal smoke dramatically decreased specific biodiversity of lung microbiome. In normal situation, it should be in a healthy balance, but inhaled carcinogens or hazardous microbes may cause cell mutations and chronic inflammation, leading to cancerogenesis. Microbiome gene expression diversity seemed to be higher than that of their human hosts because individual subjects could have different microbiomes, but all patients shared the same set of human genes, albeit frequently different alleles (26).

Recent meta-analysis using systems biology tools for a meta-transcriptome analysis of publicly available data found, among 22 differentially expressed genes, the *AKR1B10* gene from the aldo-keto reductase superfamily (AKR) which had higher expression in lung tumors in smokers than in never-smokers, and also a higher expression in tumor tissue than in normal lung tissue in never-smokers. The AKR1B10 (Aldo-Keto Reductase Family 1, Member B10) reduces aromatic and aliphatic aldehyde substrates and was shown to promote cancer cell survival. It was postulated as the initial critical step in the cascade of events leading to lung cancer (42).

Epidermal growth factor receptor, EGFR, regulates multiple cellular processes including proliferation, differentiation, and survival. Overexpression of EGFR may lead to oncogenic transformation. In NSCLC, EGFR is overexpressed in 50% to 81% of tumors (43).

Gene expression is regulated by promoter methylation of CpG islands (44); differences between smokers and never-smokers in methylation of some genes associated with smoking and lung cancer risk were recently described. Thus, Fasanelli et al. (45) found that smoking decreases methylation of several genes, particularly *AHRR* and *F2RL3*, and that their hypomethylation patterns paralleled their associations with gene expression and lung cancer risk; this was dependent on smoking status: the longer smoking cessation was, the higher the methylation of these genes and the lower the risk of cancer, approaching that of never-smokers. Subsequently, these authors found four additional CpG sites in other loci, which behaved in a similar way (46).

Multiple other reports described differences between smokers and never-smokers in promoter methylation of many genes affecting lung cancerogenesis, with sometimes conflicting results (35, 47–60). Nevertheless, cytosine hypermethylation in promoters of several genes, affecting their expression, was generally observed more frequently in never-smokers than in smokers (35) which, together with differences in gene mutations and germ-line polymorphisms (see the following sections), indicates that lung cancer in smokers and in never-smokers are two different diseases.

## 3 Gene mutations

Lung cancer in smokers and never-smokers differs also in somatic mutations occurring in cancer cells. Chromosome aberrations, e.g., loss of heterozygosity by the deletion of a chromosome segment, are more frequent in smokers than in never-smokers (35).

Mutation numbers are lower in never-smokers, but most of them are suspected to be causative for malignant transformation, whereas mutations in smokers, although more numerous, are believed to be mostly passengers without effect on the transformation induced by cigarette smoke (51–53). The tumor mutational burden was found to be more than 7 times lower in never-smokers than in smokers. Loss of heterozygosity (i.e., deletion of one allele) of human histocompatibility antigen (*HLA*) gene, encoding an antigen-presenting molecule, frequent in smokers, was rare in never-smokers (22), suggesting higher importance of specific tumor antigen presentation in smokers, consistent with a higher mutation number which produces more tumor-specific antigens. Three genetically different subtypes of lung cancer were found, based on somatic copy number variation: “piano”, dominant in never-smokers and rare in smokers, characterized by a low mutational burden, lack of somatic copy number variation, high intra-tumor heterogeneity, long telomeres, frequent *KRAS* mutations, and slow growth; “mezzo-forte”, enriched with chromosome arm-level amplifications and *EGFR* mutations; and “forte”, with whole genome doubling. *HLA* loss of heterogeneity was observed in “mezzo-forte” and “forte” types (22).

The mutation status in two genes differentiate NSCLC in smokers versus never-smokers, namely: *EGFR* and *KRAS* (see below). Epidermal growth factor receptor (EGFR) is a cell signaling molecule engaged in many cellular functions: cell proliferation, differentiation, motility, and survival. The binding of epidermal growth factor (EGF) by this receptor induces receptor dimerization, autophosphorylation and activation which initiates several molecular pathways leading to cell activation. Normally, EGFR activation ends with the exhaustion of a ligand (EGF, but also transforming growth factor alpha, TGF-α and other growth factors). However, mutations of the *EGFR* gene leading to independence from the ligand binding result in constant stimulation of uncontrolled cell proliferation and, as a result, tumor growth (54).

EGFR belongs to a family of cell membrane receptors with tyrosine kinase activity. After ligand (i.e., EGF or TGF-α in this case) binding and autophosphorylation, it transmits a signal, through a chain of intermediary molecules, among them guanine nucleotide exchange factors, to the KRAS molecule (or other RAS molecules: HRAS, NRAS), where a bound GDP is exchanged to GTP. This activates KRAS which then interacts with a multitude of effector families, initiating several pathways leading to expression of genes engaged in cell proliferation, prevention of apoptosis, actin skeleton function, transportation through Golgi, and calcium mobilization (34). Mutation in molecules (EGFR, KRAS) located at the beginning of cell activation may “fix” cellular signaling, leading to uncontrolled cell proliferation even in the absence of a ligand, and the block of apoptosis, which may result in malignant transformation.

*EGFR* mutations were associated with lung cancer in never smokers, and increasing smoke exposure was negatively correlated with mutation number (55). There are common (e.g., Ex19del and Leu858Arg) and uncommon primary mutations of the *EGFR* gene (56, 57). However, a recent finding (58) showed that only common *EGFR* mutations were more frequent in never-smokers, while uncommon single and complex mutations were more characteristic for smokers.

Other transmembrane cell-surface growth factor receptors with tyrosine kinase activity, four fibroblast growth factor receptors, FGFR1-4, activated by fibroblast growth factor (FGF) binding, also control fundamental cellular processes. Aberrations in the FGF/FGFR axis signaling may lead to many disorders, including lung cancer. In about 13% cases of lung cancer, genetic alterations (gene amplifications, mutations and rearrangements) of one of four genes from the *FGFR* family were detected, and, in patients with NSCLC, are associated with poor survival (60).

A junction of several pathways initiated by EGFR and other tyrosine kinase receptors, activated by ligand binding or mutation, is occupied by KRAS [34]. It is a GTPase, converting guanosine triphosphate (GTP) into guanosine diphosphate (GDP). When it is bound to GDP, it is inactive and does not relay signals to the cell’s nucleus. Gain-of-function *KRAS* mutations keep the protein permanently in its active state, leading to uncontrolled cell proliferation and cancerogenesis (34, 59, 61, 62).

*KRAS* mutations were found in NSCLCs (predominantly adenocarcinomas), more frequently in smokers but not in small cell lung carcinomas (32, 59, 62). In more detail, the most common *KRAS* mutations, Gly12Cys and Gly12Val, are characteristic for smokers, while other mutations appear in never-smokers, suggesting different mechanisms of carcinogenesis (61). *EGFR* and *KRAS* mutations are mutually exclusive, i.e., generally, they were not observed in the same tumor (32, 34, 61, 63). This may be explained in two ways: First, *EGFR* and *KRAS* mutations happen preferentially in never-smokers and smokers, respectively, and therefore in different individuals. Second, it seems that neoplastic cells do not need such a double mutation for uncontrolled growth; one is enough, and then there is no selection for the second one. Nevertheless, studies found a concomitant *KRAS* and *EGFR* mutation in 1.1% of NSCLC patients. However, the smoking status of these double-mutated cancers was not reported (33, 34, 64). *KRAS* mutations are usually not associated with mutations of other drivers of lung cancer development, *ALK* and *ROS1*; however, co-mutations of *KRAS* and other genes such as *TP53*, *STK11*, *KEAP1* and *NFE2L2* are more frequent (61).

*KRAS* mutations in NSCLC are more frequent (about 30% of cases) than *EGFR* mutations (15%), which is in agreement with smoking frequency in lung cancer patients. Above 80% of *KRAS*-mutated tumors display a single nucleotide variation in codon 12. Glycine to cysteine (Gly12Cys) transversion in this codon is predominant (in about 12% of patients, and making up about 40% of all *KRAS* mutations, particularly in women); other mutations (Gly12Val, Gly12Asp) are less frequent in smokers, while the rare *KRAS* mutants in never-smokers and light smokers are usually Gly12Asp (61, 62).

Tumor suppressor *TP53* mutations lead to the generation of mutant forms with altered amino acid sequences that lack DNA binding activity; they occur less frequently in tumors of never-smokers (65). Its role is described in the next section.

NSCLC genomes exhibit hundreds of nonsilent mutations together with copy number aberrations and genome doublings. De Bruin et al. (66) observed statistically significant shifts in the mutation spectra during cancer evolution. They found branched evolution, with driver mutations arising before and after subclonal diversification and pronounced intratumor heterogeneity in copy number alterations, translocations, and mutations associated with activity of APOBEC cytidine deaminases which cause predominately C→T mutations. These enzymes normally function as restriction factors of DNA-based pathogens. However, they are, unfortunately, also a significant endogenous source of genomic mutation in cancer (67, 68). They cause kataegis, which is a hypermutation confined to small genomic regions, and contribute to the large mutational burden in NSCLC. In smokers, a relative decrease in smoking-related mutations over time was observed, accompanied by an increase in APOBEC-associated mutations (66). Air pollution by fine dust such as PM10 may cause kataegis as shown *in vitro* on lung cancer cell lines (69). This may contribute to lung cancer both in never-smokers and, as an additional, synergizing factor, in smokers.

Another kind of somatic mutations in lung cancer are gene fusions. Anaplastic lymphoma kinase (ALK; see Online Mendelian Inheritance in Man, *105590) is a receptor tyrosine kinase playing a role in regulation of cell signaling and involved in the development of many cancer types, especially NSCLC. The *ALK* gene can be oncogenic by fusion with one of several other genes, by gene duplication, or by a mutation changing the genetic code. In NSCLC it may form the *EML4-ALK* fusion gene. This rearrangement is mutually exclusive with *EGFR* and *KRAS* mutations. Generally, it is detected in 3-5% of NSCLC cases (https://en.wikipedia.org/wiki/Anaplastic_lymphoma_kinase). However, in the high lung cancer prevalence Xuanwei area in China, mentioned erlier, it was found in 12.4% of NSCLC patients (7.9% men, 21.8% women). The highest *EML4-ALK* rate (35.1%, 33/94) occurred in female patients with both familial lung cancer (FLC) history and high tobacco and coal smoke exposure (25). *CD74-ROS1* fusions were less frequent than *EML4-ALK* (24, 25). The consequences of each mutation or other genetic alteration are frequently dependent on molecular changes already present in the cell. Therefore, they determine the phenotype, prognosis and response to therapy together rather than individually. A study of this broad network of functional dependence between genomic alterations requires sufficiently large and homogeneous datasets. This has been provided in the last decade by The Cancer Genome Atlas (TCGA) (70). Comparisons between cancer and non-cancer cell clones may detect the differences between benign clonal expansion and cancerous transformation (71, 72). Using only genomic features (driver genes, molecular and intratumor heterogeneity features), Chen et al. (52) divided patients evenly into three survival groups based on the predicted hazard from the multivariate Cox model. These survival models can clearly stratify patient survival outcome even within early or late stage patients, indicating the prediction power of genomic features independent of clinical features.

Thus, the differences between smokers and never-smokers in tumor mutation burden suggest again two distinct diseases, and the third one possibly in Asian women induced by a coal smoke.

## 4 Germ-line polymorphism

In addition to the somatic mutation of cancer cells, germ-line polymorphisms of the host also contribute to cancer risk.

Transcription factor p53 encoded by the *TP53* gene regulates expression of genes engaged in many cellular processes such as cell cycle, apoptosis, senescence and DNA repair. Its activity is ubiquitously lost in human cancer cells either by mutation of the *TP53* gene itself or by loss of cell signaling upstream or downstream of p53 (73, 74). *TP53* single nucleotide polymorphism c.639A>G was reported to have a statistically significant association with NSCLC (75). This variant does not induce changes in splicing (76), therefore other mechanisms should be studied.

Recently Zhang et al. (77) using large UK cohort data showed that gene polymorphisms associated with lung cancer risk have a remarkably weaker effect than smoking, particularly heavy smoking (>40 pack-years). Nevertheless, genes encoding subunits of nicotinic acetylcholine receptors were associated with the number of cigarettes smoked per day, nicotine dependence, and/or lung cancer risk (78–80).

On the other hand, gene polymorphisms affect lung cancer risk much stronger in never-smokers, where a strong effect of smoking is lacking. Suboptimal DNA repair capacity, measured by the host-cell reactivation assay, was found to be a lung cancer risk factor in never-smokers, and to synergize with the second-hand smoke effect in this group (81).

Among polymorphisms in the cytochrome P450 genes, one, Ile462Val, in the *CYP1A1* gene, is associated with lung cancer risk in never-smokers, whereas the other, Leu432Val, in the *CYP1B1* gene, carries a risk of cancer independently of smoking status. The glutathione S-transferase *GSTT1* null genotype, if combined with the *CYP1A1* variant, may confer an increased risk of lung cancer in never smokers. The Arg399Gln polymorphism of the DNA repair gene *XRCC1* was suggested to be a risk factor in never-smokers and protective in heavy smokers. Some other polymorphisms in this and other DNA repair genes were described, but sometimes with conflicting results. Chronic inflammatory lung diseases, accompanied by polymorphisms of interleukin genes *IL1* or *IL-6*, may contribute to increased risk of lung cancer in never-smokers (35).

Associations of many genetic polymorphisms with lung cancer risk were detected in Asian traditional agrarian societies where women spend long time on cooking using a coal in unventilated hearths. These polymorphisms involve immunoregulatory genes *IL1B*, *IL8RA*, *ICAM1* and *IL12A* (82), four cell cycle pathway genes *PLA2G6*, *CCNA2*, *GSK3b* and *EGF* (83) and major histocompatibility complex *HLA* class II (involved in antigen presentation to CD4+ T cells which stimulate antibody production by B cells) and *TP63* which is involved in the p53 pathway (84). A meta-analysis of publications from China, Taiwan, Japan and South Korea confirmed described earlier associations of *TP63*, *TERT*, *FOXP4*, *HLA* class II and *VTI1A* with lung adenocarcinoma in never-smoking Asian women, and detected two new associations: with *BPTF* for overall lung adenocarcinoma risk, and for *BTNL2* in cases with *EGFR* mutation. Also *HLA-DPB1* and *ROS1-DCBLD1* were associated with lung adenocarcinoma in *EGFR* mutation-positive cases stronger than in negative ones (85). It would be interesting to compare genetic associations of lung cancer in never-smoking Asian women exposed to coal smoke in rural areas with those living in urban areas without such exposure and therefore free of a strong environmental factor, be it tobacco or coal smoke. In this latter group the genetic factors should have stronger effect; however, these women may be too few to reach statistical significance.

Cancer cells may be eliminated by two types of killer cells: cytotoxic T lymphocytes recognizing, *via* T cell receptors, tumor peptides presented by class I HLA molecules (HLA-I), and natural killer (NK) cells detecting, *via* polymorphic killer immunoglobulin-like receptors (KIRs) and some other receptors, a lack of or decreased cell surface expression of HLA-I (86–88). NK-HLA-I recognition can be also disturbed by HLA-presented peptide, not allowing for successful KIR-HLA-I interaction (89, 90). Some KIRs recognize epitopes of some HLA-I molecules: thus, KIR2DL1 interacts with the C2 epitope on some HLA-C molecules, KIR2DL2 and KIR2DL3 with the C1 epitope on other HLA-C molecules, and the former interaction is stronger than the latter (91). In addition, KIR3DL1 recognizes the Bw4 epitope on some HLA-B and HLA-A molecules (92). Inhibitory KIR (such as KIR2DL1, KILR2DL2, KIR2DL3 and KIR3DL1) interaction with its ligand may protect cancer cells from NK cell-mediated killing. Indeed, Al Omar et al. (93) described in NSCLC an increased frequency of the KIR2DL1-C2 and KIR3DL1-Bw4(Thre80) combinations but decreased frequency of the KIR2DL3-C1/C1 combination, suggesting the protection of cancer cells by strong KIR-HLA interactions. In our study, we observed a decreased frequency of the C1/C2 heterozygotic genotype and increased frequencies of both C1/C1 and C2/C2 genotypes in NSCLC, suggestive of the presentation of a wider repertoire of tumor antigens to T cells by a larger panel of HLA-C molecules, or wider KIR repertoire of NK cells maturing in the C1/C2 context. In addition, patients possessing KIR2DL2 and/or KIR2DS2 and C1/C1 genotype (weaker KIR-HLA-I interactions) responded better to treatment and survived longer than patients with other genotypes (94). We also observed associations with NSCLC risk of some polymorphisms of non-classical *HLA-I* gene, *HLA-G*, and of *LILRB1* encoding an inhibitory cell surface receptor expressed by lymphoid and myeloid cells, which binds a broad range of HLA-I molecules, but preferentially interacts with HLA-G (95).

The repertoire of peptides presented by a given HLA-I molecule, or immunopeptidome, depends, first of all, on the exceptional polymorphism of HLA genes (96–99), particularly on HLA-I evolutionary divergence: the higher differences of physicochemical properties of amino acids in the peptide-binding groove between two allotypes of the same HLA class I locus, the wider the spectrum of antigenic peptides they present to CD8+ T cells, increasing the chance of an anti-tumor immune response (99).

Immunopeptidome depends also on several other molecules which contribute to antigen processing (100–103). Among these, polymorphic endoplasmic reticulum aminopeptidases ERAP1 and ERAP2 play an important role, shaping the peptide repertoire by trimming too long peptides to make them fit the HLA-I peptide-binding groove (epitope production) or overtrimming them to a length too short for HLA-I binding (epitope elimination) (102). In this way, ERAPs may influence the immune response to cancer cells. *ERAP* gene polymorphisms change activity and expression of encoded enzymes (102). We have not found any influence of particular *ERAP1* polymorphisms on the NSCLC risk in non-stratified Polish patient population (104). However, effects of these polymorphisms were revealed after stratification of patients according to smoking status: namely, they were different in smokers and never-smokers, and frequently in opposite directions (105). Thus, in this respect, NSCLC in smokers and never-smokers appears again as two genetically different diseases.

## 5 Sex differences

Men and women differ by sex chromosomes: women have two X chromosomes, while men have one X and one Y chromosome. The X chromosome is larger and contains more than 1,000 genes, whereas the Y chromosome is small and has almost four times lower number of genes. To compensate for only one X in males, one of two X chromosomes in females is inactivated stochastically in each cell. Therefore, roughly 50% of cells in a woman express the X chromosome inherited from her mother, and second 50% express the X chromosome inherited from her father. If one X has a defective allele, then only this can be expressed in a male inheriting it, while a normal allele may be expressed in nearly every second cell in a heterozygotic woman. In contrast to chromosomally determined sex, gender refers to the socially constructed roles and behaviors that influence self-identity and self-expression and is affected by social, environmental, cultural, and behavioral factors. Gender, if not identical with sex, may also affect susceptibility to some diseases by influencing human behavior (106–108). In addition, sex chromosomes influence expression of multiple genes encoded in autosomal chromosomes as well. It is not surprising then, that many diseases have different frequencies, outcome, and genetic associations in males and females – both, for example, in autoimmune diseases (109) and in cancers (110), including lung cancer (111–114).

There are multiple differences between men and women in lung cancer: men display higher smoking status in terms of both numbers of active and former smokers and numbers of smoked cigarettes; among NSCLC patients, squamous cell carcinoma is more frequent in men, and adenocarcinoma in women; higher tumor mutational burden is observed in men (112); different genes preferentially undergo mutations and other alterations in men (*TP53*, *APC*, *EPRS*, *LYST*, *KEAP1*, *STK11*, *RBM10*, *SMARCA4*) and in women (*KRAS* G12C, *ALK*, *ROS1*, *HER2*, *BRAF*) (115); men have higher PD-L1 expression while women have more frequently PD-L1-negative tumors; men have higher CD8+/CD4+ ratios and Th1 CD4+ T cells; men respond better to immune checkpoint inhibitors, while women respond better to chemo- and mutation-targeted therapy (112).

Particularly big differences in gene expression, mutations and polymorphisms between men (frequently smokers) and women (mostly never-smokers) occur in rural areas in Asia where women are continuously exposed to a smoke from coal combustion for cooking and heating, as described already in previous sections.

## 6 Ethnic differences

Most genome-wide association studies were performed on populations of European origin. In studies on non-Europeans and their comparisons with Europeans, several interesting differences were observed.

30% to 40% of Asian patients with lung cancer are never-smokers, compared with 10% to 20% of Caucasian patients (52, 59, 116). *KRAS* mutations are less frequent in never-smoking patients, and as a consequence, they are also less frequent in lung cancers from East Asia (59, 65). Genomic *ERAP1* single nucleotide polymorphism associations with NSCLC were detected in Chinese Han but not in Caucasian Poles, as mentioned already in Section 4 (Germ-line polymorphisms), before stratification according to smoking status (104, 105).

When lung adenocarcinoma patients, smokers and never-smokers, of European (EUR) and East Asian (EAS) ancestry were compared, more stable genomes with fewer genomic alterations (mutations and copy number variations) were found in EAS, and this difference was more pronounced in smokers. Consequently, EAS patients had better outcome prediction accuracy. *EGFR* mutations were much more frequent in EAS than in EUR patients. Intra-tumor heterogeneity was higher in EAS *EGFR*-mutant-bearing never-smokers than in their EUR equivalents, On the other hand, EUR patients were characterized by more frequent mutations in other driver genes, among them *KRAS*, as mentioned above. In spite of these differences, in both populations tumor mutation burden was much lower in never-smokers than in smokers (52). Concordant with results of others (9), mutations in *EGFR* and *KRAS* were mutually exclusive (52).

Further differences between EAS and EUR lung cancer patients, concerning never-smoking women inhaling coal smoke, were already described in earlier sections.

As the standard of living and medical care increases in so called third world countries, we may expect different genetic associations of lung cancer (both in smokers and never-smokers) in sub-Saharan Africans or in Latin Americans with strong native American background, who have not been so extensively examined so far.

## 7 Treatment options

Early diagnosis by screening high-risk populations using low-dose computed tomography scan and effective biomarkers may improve the survival of lung cancer patients (5, 117). In smokers, smoking cessation may decrease the chance of dying from cancer, and can help cancer treatment to work better (118–120). This shows that the effect of smoking may be reversible to some extent, and suggests that smoking cessation should be included in cancer treatment in order to improve patient survival (118, 120). In addition, an inverse association was observed between vegetable and fruit intake and lung cancer risk in current smokers, but not in former smokers and never-smokers (121).

The frequency of smoking fortunately continues to fall, at least in developed countries, while lung cancer frequency increases in never-smokers. Therefore, although this population has no such obvious strong risk factor like smokers, it should be screened for lung cancer more carefully than it was so far (19).

NSCLC is not homogenous; in contrast, there is remarkable heterogeneity within the tumor in one patient, resulting in regions differing not only in genomic variation but even in histologic type of the tumor tissue. Therefore, a biopsy limited to one region of the tumor may not give information on its nature in other regions, which may lead to unsuccessful therapy (66).

About half of never-smoking patients have mutations that may be treated using targeted therapies currently or in the near future, while potentially only 10% of ever smokers would respond to such therapy (35). Identification of targetable mutations by next-generation sequencing is important for application of directed therapy; the survival benefit of such treatment was shown to be similar in never-smokers and in former and even some actual smokers (73), although never-smokers, due to higher probability of targetable driver mutations, have better prognosis (32). A panel of small chemical inhibitors targeting *EGFR* mutations has already been elaborated and approved for medical use both in the USA and European Union. These clinical trials, which are focused on never-smoker NSCLC patients, are listed and described in detail by de Alencar et al. (32).

Due to anti-EGFR targeted therapy, patients with *EGFR* mutations survived longer than those with wild-type *EGFR* (119). However, not all *EGFR* mutations are sensitive to tyrosine kinase inhibitors (TKIs). A secondary T790M mutation arising as a response to negative selection by TKI treatment is resistant to TKIs. There are also other, less frequent mutations, called uncommon mutations, most of which are much less sensitive to TKIs than common ones. Generally, exon 20 mutations and insertions are resistant to TKIs, while mutations and deletions in other exons are more or less sensitive to this treatment (57).

Alterations of other receptor tyrosine kinases may also bring uncontrolled cell growth and cancerogenesis. One such example, as mentioned earlier (Section 4. Gene mutations), are fibroblast growth factor receptors (FGFRs). In addition to primary *FGFR* mutations, components of the FGFR pathway have also been shown to be altered in response to EGFR- or KRAS-targeted therapy as a compensatory bypass mechanism to induce drug resistance in cancer cells. Clinical trials are currently being performed to evaluate FGFR inhibitors for the treatment of lung cancers harbouring *FGFR* amplification, mutations and translocations (60, 122). Besides point mutations, fusions of the *FGFR* gene with other genes were detected in NSCLC; if the FGFR kinase domain is retained, the cell may become constantly activated, leading to malignancy (60). Alterations of the *FGFR2* gene resulting in deletion or otherwise perturbing exon 18 was recently described as a single driver of cancerogenesis. This exon encodes the C-terminal tail of FGFR2 which is proposed to moderate receptor tyrosine kinase signaling by interactions with several intracellular pathways. In contrast, other *FGFR* gene alterations, not encompassing exon 18, require co-drivers (i.e., mutations in other genes) to induce malignancy. Therefore, patients with exon 18-truncated *FGFR2* variants displayed a much better response to FGFR2 inhibitors than patients with other *FGFR2* alterations (122). Unfortunately, smoking status was not noted in this study.

Mutations of another molecule, KRAS, result in shorter overall survival and progression-free survival alongside the presence of liver and brain metastases. About half of *KRAS* mutant NSCLC cases possess concomitant mutations in other genes (*TP53*, *STK11*, *KEAP1*), which may be reflected in different (better or worse) response to treatment. Updated reviews concerning the therapy of *KRAS* mutant lung cancers are given in detail by Ceddia et al. (59) and Cekani et al. (61). As the Gly12Cys mutation of *KRAS* is most frequent (particularly in smokers), the design of its inhibitors targets the mutated cysteine residue as a covalent tether to bind to KRAS in the so called Switch II pocket. Oxidation of this cysteine residue blocks covalent binding of KRAS Cys12 inhibitors. Therefore, assessment of KRAS Cys12 oxidation status in tumors prior to treatment may facilitate finding the optimal therapy for patients with this mutation (123). New computational methods enable the determination of receptor molecular structure and flexibility in order to design direct inhibitors of KRAS Cys12 (124). However, secondary mutations, arising after treatment of KRAS Cys12 mutant with specific inhibitors such as sotorasib and adagrasib, may confer resistance to these inhibitors. Other components of the MAPK pathway, downstream from KRAS, may also cause such resistance (62, 125). Results of an *in vitro* study on mutated cell clones suggest one possible strategy to overcome such acquired resistance (126). Oncogenic KRAS induces feedback inhibition of wild-type RAS signaling. Therapeutic inhibition of oncogenic KRAS disengages this negative feedback pathway, leading to wild-type RAS activation and triggering adaptive drug resistance. This may be overcome by attacking either upstream or downstream molecules in the KRAS pathway. Many clinical trials are currently underway to achieve this goal (125), It is, therefore, a matter of time to see whether smokers and never-smokers respond differently to such treatment.

## 8 Immunology and immunotherapy

Lungs are one of the places (together with the skin, gastrointestinal tract and urogenital tract) where our body comes into contact with external factors, some of which may be harmful. Therefore, the recognition of foreign substances and microbes and their elimination is needed. Several types of antigen-presenting cells (dendritic cells, macrophages, but also epithelial and endothelial cells) activate antigen-specific CD8+ and CD4+ T cells in the lung; their subpopulations and functions have recently been described in detail by Kawasaki et al. (127). The effect of smoking, air pollution and other factors on the immune system, leading to changes in abundance and activity of particular immunocyte subpopulations in the lung and to higher risk of lung cancer is reviewed deeply in great detail in our Research Topic “Comparison of lung cancer and chronic obstructive pulmonary disease in smokers and never-smokers” by Taucher et al. (21) and de Alencar et al. (32).

The immune system evolved to defend the organism against foreign pathogens as well as against arising neoplastic cells, but it must be tolerant to healthy self cells and their structures (86, 87). Central tolerance is established by deleting autoreactive T and B lymphocyte clones during their differentiation in the thymus and bone marrow, respectively, before they develop into fully immunocompetent cells (128–130). Peripheral tolerance, preventing activation of these autoreactive clones which escaped from the central tolerance mechanism, is provided by regulatory T cells (Tregs) suppressing antigen-specific responses (131), as well as by immune checkpoints (132). These latter molecules perform their regulatory function by interacting with their receptors on activated T cells and inhibiting their effector functions. The physiological role of immune checkpoints is to prevent the organism from anti-self response which could lead to autoimmune disease or exaggerated immune response to innocent antigens. However, they may also be used by cancer cells to directly suppress host immune responses (32, 132–134). Four years ago, the Nobel Prize in Physiology or Medicine was awarded to James Allison and Tasuku Honjo, discoverers of Cytotoxic T-Lymphocyte-Associated protein 4 (CTLA-4) and Programmed Death 1 (PD-1) protein, respectively. Since the time of their discoveries, a new direction in cancer immunotherapy, namely the use of immune checkpoint inhibitors, has developed and achieved great success (135–137). However, lung adenocarcinomas in never-smokers respond poorly to immune checkpoint inhibitors; due to a lower number of mutations they do not produce many neoantigens, but secrete many immunosuppressive factors, and the tumor microenvironment is non-permissive (32).

A meta-analysis of anti-PD-1 and anti_PD-L1 treatment of NSCLC patients supplemented or not with chemotherapy showed greater effect of anti-PD-1 alone in men, whereas women benefitted better from anti-PD-1 and anti-PD-L1 together with chemotherapy (112). Even in patients of both sexes selected for high PD-1 expression on their NSCLC cells, the effect of anti-PD-1/anti-PD-L1 monotherapy was much stronger in men than in women who required chemotherapy additionally to achieve similar benefit (112). Unfortunately, in all studies included in these analyses, never-smokers were a small minority or were excluded, so the effect of smoking could not be assessed here. Therefore, the lack of a large contribution of never-smokers may explain why the immune checkpoint inhibition monotherapy was effective, at least in men.

KRAS mutants express higher levels of the PD-L1 molecule and attract T cell infiltration, which is important for immunotherapy (34, 61).

The immune response to tumor antigens and the result of immunotherapy depends, among other factors, on the diversity of *HLA* alleles of the patient. Effect of *HLA-I* evolutionary divergence on immunotherapy was first described in metastatic melanoma and NSCLC (99). Increased sequence divergence of *HLA-I* alleles was associated with increased diversity of self, tumor and viral immunopeptidomes. Patients with both high tumor mutational burden (frequently smokers) and high *HLA-I* evolutionary divergence responded better to immune checkpoint inhibitors than those with lower *HLA-I* allele divergence (99).

Immune surveillance mechanisms aiming at elimination of cancer cells may result in immunoselection, i.e., survival of non-immunogenic clones, not recognized by the immune system. This may be achieved by cancer cells directly by mutation of genes encoding epitopes recognized by CD8+ T cells and by mutation of *HLA-I* genes or antigen-presenting machinery genes, or indirectly by epigenetic silencing of *HLA-I* and antigen-presenting machinery gene expression. Endoplasmic reticulum aminopeptidases mentioned in Section 4 (Germline polymorphisms) are important elements of antigen-presenting machinery, and their polymorphisms affect their expression and activity (102). ERAPs and other antigen-presenting machinery molecules may enhance or destroy presentation of tumor antigens to CD8+ T cells (138). Targeting ERAPs with small molecular inhibitors can be potentially used in therapy of many diseases including cancer (138–140).

Burr et al. (141) showed a transcriptional silencing of both *HLA-I* and antigen-presenting machinery genes by polycomb repressive complex 2 in small cell lung cancer. This histone methyltransferase plays a physiological role in stem cell differentiation and early embryonic development, but is also sometimes used by cancer cells to avoid eradication by the immune response. This process is potentially reversible and might become a target of cancer therapy (142).

Combinations of EGFR-tyrosine kinase inhibitors with anti-vascular endothelial growth factor monoclonal antibodies in NSCLC patients with different *EGFR* mutations gave inconsistent results so far (31). In patients with the *EGFR* T790M mutation, anti-vascular endothelial growth factor monoclonal antibody (bevacizumab) with a covalent third-generation EGFR-tyrosine kinase inhibitor (osimertinib) failed to show prolongation of progression-free survival and overall survival compared with osimertinib alone (142). A comprehensive review of the latest reports on lung cancer immunotherapy in never-smokers is given by de Alencar et al. (32).

The tumor microenvironment, consisting of several cell types, plays a significant role in immune evasion, resistance to therapy and the promotion of malignancy; this has been recently described in detail by Khalaf et al. (143).

Lung cancer research in the past has long focused on smokers, as smoking has been an obvious strong risk factor since 1950 (13–15). Such studies in never-smokers are delayed, but with growing percentages and numbers of lung cancer cases in never-smokers the data started to accumulate. With growing knowledge of genetic and non-genetic factors contributing to this disease, we may suppose in future a personalized treatment of each patient, based on his/her genetic background and environmental exposure.

## 9 Conclusion

Multiple features differentiate lung cancer in smokers and never-smokers, suggesting that they are two different diseases, and smokers suffer from this cancer much more frequently than never-smokers. Inhalation of coal smoke during cooking and heating in some areas carries additional risk of lung cancer, even in never-smokers. Altogether, over 2 million people in the world are diagnosed with lung cancer every year, most of whom die, unfortunately. However, the world population recently approached 8 billion. Therefore, new lung cancer patients make only 0.025% of the total human population. Our organisms are protected against lung cancer by multiple immune and non-immune mechanisms. Although “even monkeys fall from trees”, but only some of them; most monkeys persist on tree branches, and most humans are free from lung cancer, although their activities (smoking, air pollution etc.) increase the probability of suffering from, like monkeys jumping from branch to branch and from tree to tree increase the probability of falling. Some of us, unfortunately, have a combination of unfavorable alleles of genes predisposing us to lung cancer; they may receive targeted treatment which has improved in recent years together with the progress of science. But they and also others should not artificially increase their risk of lung cancer by smoking and non-ecological behavior both individually and as a society. It depends on our wisdom whether we increase or decrease cancer risk by changing our way of life and ecosystem.

## Author contributions

The author confirms being the sole contributor of this work and has approved it for publication.
